# Functional Dissociation of the Posterior and Anterior Insula in Moral Disgust

**DOI:** 10.3389/fpsyg.2018.00860

**Published:** 2018-06-01

**Authors:** Xiaoping Ying, Jing Luo, Chi-yue Chiu, Yanhong Wu, Yan Xu, Jin Fan

**Affiliations:** ^1^Beijing Key Laboratory of Applied Experimental Psychology, National Demonstration Center for Experimental Psychology Education, Faculty of Psychology, Beijing Normal University, Beijing, China; ^2^Institute of Sociology, Chinese Academy of Social Sciences, Beijing, China; ^3^School of Psychology, Capital Normal University, Beijing, China; ^4^Department of Psychology, The Chinese University of Hong Kong, Shatin, Hong Kong; ^5^School of Psychological and Cognitive Sciences, Peking University, Beijing, China; ^6^Department of Psychology, Queens College, The City University of New York, Flushing, NY, United States; ^7^Department of Psychiatry, Icahn School of Medicine at Mount Sinai, New York, NY, United States; ^8^Fishberg Department of Neuroscience, Icahn School of Medicine at Mount Sinai, New York, NY, United States; ^9^The Friedman Brain Institute, Icahn School of Medicine at Mount Sinai, New York, NY, United States

**Keywords:** moral disgust, agent, posterior insula, anterior insula, fMRI

## Abstract

The insula is thought to be involved in disgust. However, the roles of the posterior insula (PI) and anterior insula (AI) in moral disgust have not been clearly dissociated in previous studies. In this functional magnetic resonance imaging study, the participants evaluated the degree of disgust using sentences related to mild moral violations with different types of behavioral agents (mother and stranger). The activation of the PI in response to the stranger agent was significantly higher than that in response to the mother agent. In contrast, the activation of the AI in response to the mother agent was significantly higher than that in response to the stranger agent. These data suggest a clear functional dissociation between the PI and AI in which the PI is more involved in the primary level of moral disgust than is the AI, and the AI is more involved in the secondary level of moral disgust than is the PI. Our results provide key evidence for understanding the principle of embodied cognition and particularly demonstrate that high-level moral disgust is built on more basic disgust via a mental construction approach through a process of embodied schemata.

## Introduction

Morality is the center of our attitudes and behaviors in daily social life and beyond (Haidt and Kesebir, 2010). Moral judgment has been generally recognized to encompass not only reasoning but also emotion and affection (Greene and Haidt, 2002), and disgust has a strong impact on moral judgment and is rudimentary to moral emotion (Miller, 2008; Rozin et al., 2008; Giubilini, 2016). Neuroimaging studies have shown that the insula is involved in physical disgust, moral judgment (Moll et al., 2005), and detecting norm violations (Xiang et al., 2013; Cheng et al., 2017). However, how moral disgust is encoded and represented in the insula remains unclear.

The insular cortex, which is a key region responsible for encoding and re-encoding feelings, consists of regions with variable cell structures or cytoarchitectures ranging from granular in the posterior portion to agranular in the anterior portion (Flynn, 1999; Varnavas and Grand, 1999). The posterior-to-anterior progression, which includes increasingly complex representations in the human insula, indicates that the posterior insula (PI) plays a role in encoding more primary emotions, the mid-insula plays a role in encoding contextual integration (Craig, 2002, 2009), and the anterior insula (AI) plays a role in encoding introspective awareness of emotion and bodily states (Critchley et al., 2004; Paulus and Stein, 2006). This hypothesis provides a new perspective for understanding how a complicated, high-level mentality or emotionality is built or developed from more basic feelings.

A neuroimaging study investigating the relationship between love and sexual desire revealed that the anterior part of the insula was significantly activated by feelings of love, whereas the posterior part of the left insula was significantly activated by primary feelings, such as sexual desire (Cacioppo et al., 2012). A study investigating the neurodevelopmental changes in the circuits underlying empathy and sympathy from childhood to adulthood found a significant negative correlation between age and the degree of activation in the PI and a positive correlation in the anterior portion of the insula (Decety and Michalska, 2010), suggesting that a higher level of frontalization of inhibitory capacity and a greater top–down modulation of activity occur in primitive emotion-processing regions during individual development (Yurgelun-Todd, 2007). In a study investigating fairness in relation to moral judgments, the PI was selectively associated with the processing of the objective aspects of fairness, whereas the more anterior part, i.e., the mid-insula, was involved in the processing of the contextual aspects of fairness, suggesting that the mid-insula performs a re-encoding function for the integration of context with inequality (Haidt and Kesebir, 2010; Wright et al., 2011).

However, studies investigating the involvement of the anterior and posterior insula in moral judgment and disgust have been inconsistent. Most studies report that the anterior part of the insula was activated in moral indignation/disgust relative to pure disgust (Moll et al., 2005), while passively viewing pictures depicting social moral violations relative to viewing these pictures with an endeavor to decrease emotional reactions (Harenski and Hamann, 2006), in deontological guilt relative to altruistic guilt (Basile et al., 2011), while retrieving personal guilt or shameful memories (Wagner et al., 2011), and in guilt associated with prejudice (Fourie et al., 2014). In addition, compared with the processing of easy moral dilemmas, the anterior part of the insula was involved in the processing of difficult personal moral dilemmas (Greene et al., 2004) and difficult dilemmas where the to-be-sacrificed person was humanized as a full-blown individual with mental states (Majdandžić et al., 2012). In contrast to the involvement of the anterior part of the insula, the involvement of the PI in moral processing, such as in a comparison between moral indignation and a neutral condition (Moll et al., 2005) or between sociomoral violation actions and physically repulsive actions (Schaich Borg et al., 2008), has only been occasionally reported.

To date, no study has doubly dissociated the function of the PI and AI in moral disgust. Given that functional segregation has been generally established in this extensive and cytoarchitectonically diverse cortical region (Flynn, 1999; Varnavas and Grand, 1999), the double dissociation of the PI and AI in moral disgust could have important theoretical implications for moral cognition and emotion, particularly for the theory that disgust in response to moral violations is built on more basic types of disgust (such as that associated with distaste for food and body waste products) through a process of embodied schemata, which refers to patterns of experience that are based on bodily knowledge or sensation (Haidt et al., 1997).

In this study, we separated the following two components involved in the representation of moral disgust: the primary moral disgust component represented in the PI and the secondary component represented in the AI. The dissociation of these two components was achieved by requiring participants to process moderate moral transgression behaviors (e.g., speaking loudly on the telephone in a public place or saying dirty words in a public place) with different behavioral agents (stranger or mother). Moral indignation toward a stranger who behaves immorally was relatively primary and featured feelings of anger and hate; thus, the PI was challenged. However, moral indignation toward the mother was relatively secondary, required relatively high levels of integration and regulation and featured feelings of shame or guilt; thus, the AI was involved.

## Materials and Methods

### Participants

Thirty-six healthy, right-handed students with normal or corrected-to-normal vision participated in this study. No participants had a history of neurological or psychiatric disorders or head injury. Of these participants, six participants were excluded from fMRI analysis due to device or technical errors, and one participant was excluded due to excessive head movement (>3 mm). The final sample included 29 participants (14 females; mean age 22.4 ± 2.40; range 19–28 years). The participants were compensated for their time. Before the fMRI scan, written informed consent approved by the local Ethics Committee at Beijing Normal University was obtained from each participant. Before the fMRI experiment, we asked the participants to answer a list of self-developed questions regarding their relationship with their mothers, and only individuals who indicated a close relationship with their mothers [quantified by answering a 4 or 5 on a five-point Likert scale from 1 (“very bad”) to 5 (“very good”)] were included in the sample. The participants also completed the Chinese version of the Yale-Brown Obsessive Compulsive Scale (Goodman et al., 1989) and Toronto Alexithymia Scale (Parker et al., 2003).

### Materials

The experimental stimuli included 60 sentences describing situations of moral transgressions (e.g., speaking loudly on the telephone in a public place) that frequently occur in daily life and reliably evoke moral disgust. The severity of the moral transgressions was controlled at a moderate level because a serious transgression (such as “killing someone”) was inapplicable to the participants’ mothers. The length and complexity of the sentences were carefully controlled. Another sample of participants (*N* = 100) who did not participate in the formal fMRI experiment rated the severity of the moral transgressions and the emotional arousal associated with each moral transgression event. The severity of the moral transgressions was rated using a six-point Likert scale from 1 (“not serious at all”) to 6 (“extremely serious”), and five emotions, i.e., disgust, anger, surprise, sadness and disappointment, were rated using a seven-point Likert scales from 0 (“no feeling at all”) to 6 (“extremely strong”). The 60 sentences had a similar length and complexity and were divided into three equal groups according to the severity and emotional arousal ratings. No statistically significant differences were observed in the severity of the moral transgressions, moral disgust or other types of emotional arousal among the three groups of materials (**Supplementary Table S1**). In the fMRI experiment, each group of materials was assigned to only one of the three experimental conditions that used the “stranger,” “mother,” or “best friend” as the behavioral agent (**Table 1**). The assignment of a given agent to a given group of materials were counter-balanced across the participants.

**Table 1:** Sample sentences from the experimental materials.

	Stranger	Best friend	Mother
Moral disgust	Stranger says dirty words in a public place	Best friend chats at a concert	Mother speaks on the telephone loudly in a public place

### Task Design and Procedures

**FIGURE 1:**
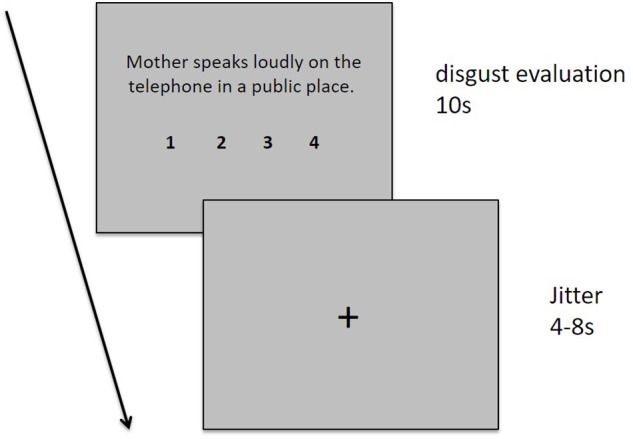
Task design. Each sentence was displayed for 10 s, followed by the presentation of a fixation cross for 4–8 s. A fixation cross was presented for 30 s at the start and end of the task and for 16 s during each trial.

During the experimental fMRI scan session, the participants were asked to read and evaluate 60 sentences describing different moral transgression events with different behavioral agents (mother, stranger, and best friend) one-by-one. Each sentence was presented for 10 s, followed by a cross fixation phase of a varied duration ranging from 4 to 8 s. During the 10-s sentence presentation stage, the participants were instructed to read and comprehend the situation described by the sentence and evaluate their degree of disgust using a four-point Likert scale from 1 (“not disgusting at all”) to 4 (“extremely disgusting”). The participants were required to indicate their evaluation by pressing one of four buttons using their index, middle, fourth, or little finger of their right hand. The degree of disgust and the numbers 1, 2, 3, and 4 were presented below the sentence (see **Figure 1** for a detailed description of each sentence).

To prevent the participants from frequently switching between the behavioral agent of the moral transgressions, 20 sentences in each condition were separated into two sub-groups with 10 sentences in each sub-group, and the 10 sentences in each sub-group were presented successively in one block. Therefore, two blocks of each of the three experimental conditions involved the mother, stranger and best friend as the behavioral agent, and the participants completed a total of six blocks. The sequences of the block presentations were counter-balanced across the participants with the restriction that two blocks in the same condition could never be presented successively. During the periods between the blocks, a fixation (cross-viewing) was presented for 16 s. In addition, 30-s fixation periods were presented at the beginning and end of the session.

### Image Acquisition

All MRI scans were acquired using a Siemens MAGNETOM Trio 3T MR scanner at the Imaging Center for Brain Research at Beijing Normal University. Foam padding and a plastic brace were used to minimize head movement. For the functional imaging, the whole-brain coverage of 33 axial slices was acquired using a T2-weighted echo-planar imaging sequence based on the blood oxygenation level-dependent (BOLD) contrast with the following parameters: 2000 ms repetition time (TR), 30 ms echo time (TE), 90° flip angle, 4.0-mm slice thickness, 0.6-mm gap, 64 × 64 data matrix, 200-mm field of view (FOV), and 3.1 × 3.1 × 4.0-mm voxel size. In addition, 3D structural brain scans were also acquired for each participant using a T1-weighted anatomical scan with the following parameters: 2530-ms TR, 3.39-ms TE, 7° flip angle, 256 × 256 data matrix, 256-mm FOV, 1.3 × 1.0 × 1.3-mm voxel size, and Bandwidth (BW) = 190 Hz/pixel.

### Image Data Analysis

The event-related analyses of the fMRI data from the moral disgust task were conducted using a statistical parametric mapping package (SPM8; Wellcome Trust Centre for Neuroimaging, London, United Kingdom). In the preprocessing of the data, each image volume was slice-time corrected, realigned, unwarped to the first volume, co-registered to the structural scan images, spatially normalized to the Montreal Neurological Institute (MNI) ICBM152 space based on the normalization parameters of the T1 image, subsampled to a voxel size of 2 × 2 × 2 mm, and finally spatially smoothed using a Gaussian kernel of 8 mm full-width half-maximum.

For statistical analysis, a general linear model (GLM) was constructed to analyze the functional scans from each participant with a duration of 10 s by regressing the observed event-related BOLD signals on the regressors to identify the relationship between the hemodynamic responses and task events. Low-frequency drifts in the signal were removed using a high-pass filter with a 128-s cutoff. Regressors were created by convolving a train of delta functions representing the sequence of individual events using the default SPM basis function, which consists of a synthetic hemodynamic response function (HRF) composed of two gamma functions (Friston et al., 1998). Three regressors were used for the three conditions (mother, best friend and stranger). The 6 parameters generated during the motion correction were also entered as covariates. In addition, HRF related to trials in which the participants failed to respond was also modeled separately and explicitly to partial out error-related activity. Linear contrasts of the parameter estimates were performed to identify the effects of the three conditions and the difference between every two conditions in each session. Then, the first level contrasts were aggregated into a second level, and one-sample *t*-tests were performed to compute the group-level statistics using a random-effects model.

#### Regions of Interest (ROIs) and Psychophysiological Interaction (PPI) Analysis

To define the regions of interest (ROIs), we first conducted contrasts between the stranger condition and the mother condition. To test our hypotheses regarding the role of the AI and PI in moral disgust, ROI analyses were performed based on the templates developed by Lin and colleagues (Lin et al., 2013), which consisted of six insula regions, including the left and right AI (LAI and RAI), left and right PI (LPI and RPI), and left and right middle insula (LMI and RMI). The ROIs were defined by masking the six abovementioned insula regions on the whole brain results of a given contrast (e.g., the contrast of “mother condition minus stranger condition” and the contrast of “stranger condition minus mother condition”). The significance level was set at an uncorrected threshold of *p* < 0.05 with a cluster extent of at least 5 contiguous voxels. The LAI was activated in the mother condition minus the stranger condition. The LPI was activated in the stranger condition minus the mother condition (see **Table 2** for details). ROIs as clusters were created for the LAI and LPI. The BOLD signal changes were extracted from each ROI for the contrast between the stranger condition and the mother condition. Separate psychophysiological interaction (PPI) analyses were also performed using the LAI or LPI ROIs as seeds.

**Table 2:** Brain activation of the insula in a contrast between the stranger and mother conditions.

Insula region	Side	*x*	*y*	*Z*	*T*	*Z*	*P*	*K*
**Moral disgust: Mother > Stranger**					
Anterior	Left	-34	18	-16	1.83	1.77	<0.05	43
**Moral disgust: Stranger < Mother**					
Posterior	Left	-38	-10	22	2.28	2.17	<0.05	66
		-42	-8	12	2.22	2.12	<0.05	
middle	Right	42	2	0	2.02	1.94	<0.05	18

*P < 0.05, *K* > 5 of 2 mm × 2 mm × 2 mm voxels, LAI (-34 18 -16), LPI (-38 -10 22), RMI (42 2 0)*.

Psychophysiological interaction analyses provide a measure of functional connectivity change among different brain regions depending on a specific psychological context (Friston et al., 1997). This analysis was achieved using a moderator derived from the product of the activity of a source region and the psychological context. The LAI and LPI were derived from the ROI analysis and identified in moral disgust by the saliency level of the contrast between the stranger condition and the mother condition (see the Results). We aimed to determine whether the AI and PI functionally interact with regions involved in secondary and primary moral disgust processing, respectively. PPI analysis was performed to identify the region(s) that had differential connectivity with the AI and PI modulated by the difference between the stranger agent and the mother agent in moral disgust.

## Results

Before presenting the results, the following two points should be noted. First, in this paper, we focused on the results of the mother condition and stranger condition, and the results of the best friend condition are not reported in the present paper. This condition was omitted because the main goal of this study is to dissociate the function of the PI and AI in moral disgust. The ideal way to achieve this goal is to perform a direct contrast between a very intimate relationship, i.e., the mother condition, and a very distant relationship, i.e., the stranger condition. Furthermore, the best friend condition complicates the situation because the nature of friendship is unclear and could vary from person to person (**Supplementary Figure S1**). Second, regarding the brain imaging results, we only focused on the ROIs in the anterior, middle and PI and brain regions found to be functionally connected to these ROIs. We conducted and inspected the results of the whole-brain analysis and confirmed the general validity of the results. For example, we confirmed that the brain activation we observed during the visual, linguistic, and cognitive control processing in our moral judgment task was similar to that reported in other related studies. However, these results are not the focus of this study and are provided in the Supplementary Materials.

### Behavioral Results

**FIGURE 2:**
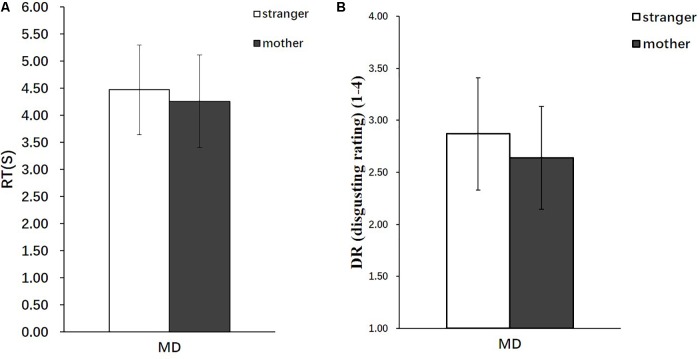
Behavioral results (*n* = 29). **(A)** RT in the stranger condition and mother condition. **(B)** DR in the stranger condition and mother condition. Error bars represent the SD.

The behavioral data analyzed included the disgust ratings (DR), response times (RT), and severity ratings (SR). The online recording of the participants’ behavioral responses during the MRI scanning showed that the participants required a significantly longer duration to complete the DRs in the stranger condition [Mean = 4.47, standard deviation (SD) = 0.824] than in the mother condition (Mean = 4.26, *SD* = 0.854) [*t*_(28)_ = 2.55, *p* < 0.05] (**Figure 2A**), and the participants rated the strangers performing moral transgressions as significantly more disgusting (Mean = 2.87, SD = 0.539) than those of the mothers (Mean = 2.64, *SD* = 0.494) [*t*_(28)_ = 2.76, *p* < 0.01] (**Figure 2B**).

### fMRI Results

#### ROI Analysis

**FIGURE 3:**
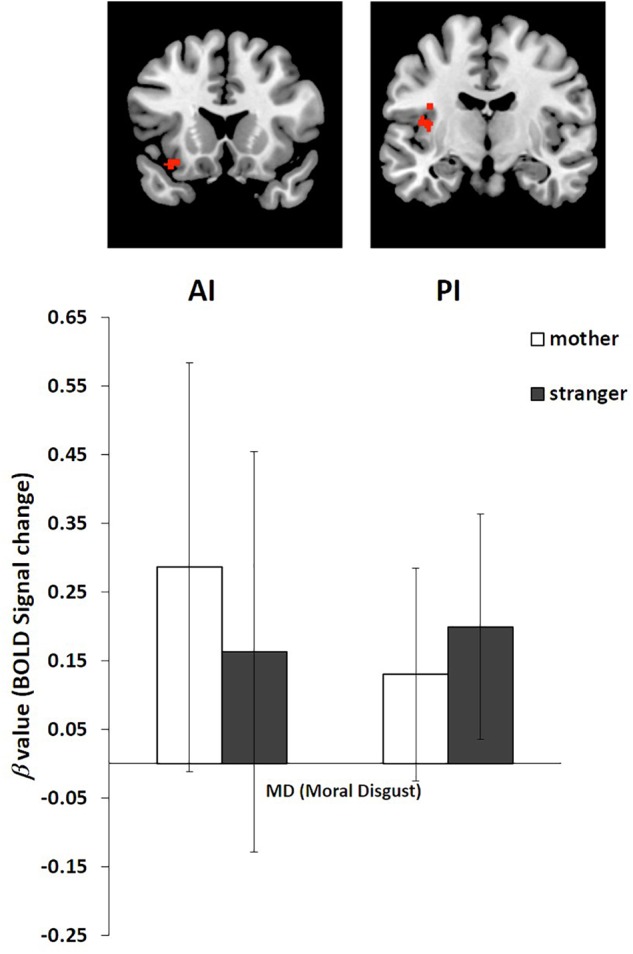
Regions of interest (ROI) analysis of BOLD signal change in the AI and PI in the stranger condition and mother condition. Error bars represent the SD.

We performed an ROI analysis of the clusters of the AI and PI based on the activation of these two regions in the stranger condition and the mother condition of moral disgust (**Figure 3** and **Table 2**). The coordinates of the ROIs were as follows: AI, the center of the cluster at [-34 18 -16], and PI, the center of the cluster at [-38 -10 22].

The β value of the mother and stranger conditions were extracted from the AI and PI ROIs. For the AI, the β value in the stranger condition (Mean = 0.16, *SD* = 0.292) was lower than that in the mother condition (Mean = 0.29, *SD* = 0.298). For the PI, the β value in the stranger condition (Mean = 0.20, *SD* = 0.164) was greater than that in the mother condition (Mean = 0.13, *SD* = 0.155) (**Figure 3**).

### PPI Analysis

**FIGURE 4:**
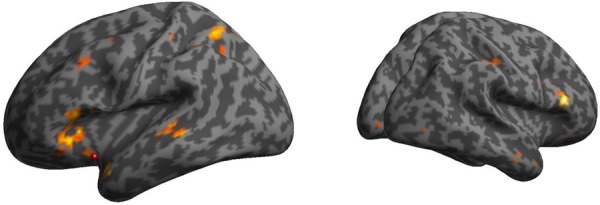
Psychophysiological interactions (PPIs) of the AI in the mother condition minus stranger condition. The bilateral prefrontal cortex was functionally connected with the AI in the mother condition minus stranger condition.

The PPI of the AI and PI seeds represents how the agent (mother/stranger) modulates the change of the connectivity between the seeds regions and other brain regions. In the mother condition, the AI was more functionally connected with bilateral prefrontal cortex (PFC) relative to the stranger condition (**Figure 4**), whereas in the stranger condition, the PI was more functionally connected with thalamus and amygdala, the AI was more functionally connected with anterior cingulate cortex (ACC), and both PI and AI were more functionally connected with temporo-parietal junction (TPJ), relative to mother condition (**Table 3**).

**Table 3:** Significant PPIs of the AI and PI seeds.

Region	*x*	*y*	*z*	*T*	*Z*	*K*
***AI: Positive PPI***					
R insula	36	-16	22	2.93	2.71	34
	46	4	-10	2.36	2.24	56
L TPJ	-34	-38	30	3.61	3.25	109
R TPJ	64	-30	32	2.99	2.76	600
R ACC	18	32	20	3.81	3.39	68
	2	28	-2	2.31	2.19	111
L ACC	-2	32	20	2.17	2.07	71
***AI: Negative PPI***					
L insula	-26	24	4	2.8	2.61	104
	-34	24	-4	2.48	2.34	
***PI: Positive PPIs***					
R thalamus	16	-16	18	3.14	2.88	99
R TPJ	40	-32	40	2.66	2.49	191
R amygdala	22	-2	-14	22	-2	69
L caudate	-8	20	4	2.89	2.68	160
R caudate	10	16	12	2.42	2.29	125
L ACC	-20	32	24	2.57	2.42	48

*P < 0.05, uncorrected, 2 mm × 2 mm × 2 mm voxels. TPJ, temporo-parietal junction. ACC, anterior cingulate cortex*.

## Discussion

The behavioral results indicated that the RTs in the mother condition were quicker than those in the stranger condition, which could be due to people devoting less time to thinking negative thoughts about their mother because these thoughts may evoke strong unpleasant feelings and the desire for avoidance (Li et al., 2011). Unsurprisingly, the moral transgressions performed by the mother were rated as less disgusting and less severe than those performed by the stranger. This bias could be related to the participants’ internal tendency to favor their mothers in moral judgments. In particular, in our Chinese participants who may mentally represent themselves and their mothers by the same cognitive-brain mechanism (Zhu et al., 2007), this bias could be more obvious (Hwang, 2006).

Critically, the brain imaging results exhibited a double dissociation between the AI and PI in which an AI activation was found in the mother condition minus stranger condition contrast, while a PI activation was found in the stranger condition minus mother condition contrast. Furthermore, PPI analysis indicated that these PI and AI areas were functionally connected to widely distributed areas, including areas that are necessary for the representation of sensations and feelings, emotion regulation, and theory of mind (ToM).

### The Role of the AI in Moral Disgust

Relative to the stranger condition, the mother condition was associated with AI activation. Similar activation was reported among people who were required to recall personal guilt experiences (Shin et al., 2000) or internally generate deontological guilt (Basile et al., 2011). Generally, the role of the ventral AI observed in this study has been proposed to mediate the core affect representing broadly tuned motivational states (e.g., excitement) with associated subjective feelings (Wager and Barrett, 2004).

Several hypotheses could be applied to explain why the mother condition was associated with more AI activation than was the stranger condition. For instance, making a moral judgment in the mother condition could require more integrative processing. AI activation could be related to the feelings that are represented on a more integrative level relative to the less integrative level, such as the feeling of love relative to sexual desire (Cacioppo et al., 2012) or the processing of the contextual aspects of fairness relative to the processing of objective aspects (Wright et al., 2011). Compared with making a bad moral judgment against a stranger, making a judgment against one’s mother could involve more conflict of self-interest. Learning to make moral judgments based on considerations beyond self-interest is a fundamental aspect of moral development that can be achieved by communicating and interacting with many more people than one’s parents or the process of deliberate (moral) persuasion intentionally generated by certain people to vividly demonstrate their value judgment (Bloom, 2010). Therefore, more integrative processing could be required by this type of moral judgment. A second possibility regarding the involvement of the AI in the mother condition could be the requirement for more processes of emotion regulation. A previous study found that the development of emotion regulation capacity with age could be accompanied by a posterior-to-anterior progression in insula activity in response to empathy- or sympathy-eliciting stimuli (Decety and Michalska, 2010). In this study, more emotion regulatory processes could be required in people evaluating their mothers’ immoral behaviors than thinking of a stranger performing the same behaviors. Third, according to a previous study that found that the AI could function together with other prefrontal and temporal-parietal areas as a mechanism of “guilt aversion” that motivates people to choose to cooperate if they can better serve their interests by acting selfishly (Chang et al., 2011), the mother condition could contain more components of “guilt aversion” because of the close relationship with the mother. This closeness might result in stronger AI activation. Finally, in the moral context, the AI was found to be selectively activated in negative moral verdicts that identified an act as morally wrong regardless of whether the acts transgressed against moral principles more or less or required more or less moral deliberation (Schaich Borg et al., 2011). This finding is generally consistent with previous studies indicating that the activity in the AI correlated with the rejection of unfair offers (Sanfey et al., 2003), rejection of inequitable allocations (Hsu et al., 2008), decisions not to donate to charity (Moll et al., 2006), decisions not to purchase in a shopping task (Knutson et al., 2007), and verdicts of disbelief (Harris et al., 2008). Although the participants in our study made comparable negative moral verdicts in both conditions, making a fair judgment in the mother condition might require more resolution and extensive processing of negative moral verdicts and, thus, evoke more AI activation.

### The Role of PI in Moral Disgust

Relative to the mother condition, the stranger condition was associated with PI activation. The involvement of the PI in moral-related tasks has been much more rarely reported than that of the AI. The PI is known to function in primary representations of emotionally relevant somato-sensory signals (Craig, 2002), such as primary pain, temperature, and touch perception, including facilitative touch (Björnsdotter et al., 2009; Löken et al., 2009; Lamm et al., 2011). Studies have reported PI activation in participants reading phrases that elicited moral indignation compared to that in participants reading neutral phrases (Moll et al., 2005) and in participants processing sociomoral acts (the immoral ones) compared to participants processing pathogenic acts (physically repulsive ones) (Schaich Borg et al., 2008). Notably, the examples of moral violations used in the Borg and colleagues’ study were more serious than the mild violations used in the present study. In the previous study, the materials that the participants read included statements, such as “You watching your sister masturbate” or “You killing your sister’s child.” A study investigating major depressive disorder (MDD), including excessive proneness to self-blaming emotions, such as guilt and shame, exhibited an increasing PI activation in response to shame relative to that in response to guilt, implying that the specific function of PI in generating moral disgust related feeling (Pulcu et al., 2014). Additionally, PI activation was observed in social rejection, particularly when the rejection is powerfully elicited (Kross et al., 2011). An intracranial electroencephalography study found that, in contrast to the AI, which showed an initial fast response to social exclusion with a rapidly fading signal, the PI showed a more persistent activation pattern, implying that the PI represents a more primary aspect of disgust that does not decay over time (Cristofori et al., 2013). In addition to social rejection, PI activation was also observed in the processing of unfair offers in the ultimatum game, and its activation level could be modulated by emotion regulation strategies (Kirk et al., 2011), although the AI is much more frequently reported to be involved in the ultimatum game (Rilling et al., 2002; Sanfey et al., 2003; King-Casas et al., 2008). Both the social rejection in the Cyberball task and the unfair offers in the ultimatum game could be types of moral violations due to their nature, and the participants are the victims of these immoralities. This type of deep and painful feeling could eventually evokes a body sensation-like PI activation. In the present study, the participants likely perceived themselves as the victims of moral violations more in the stranger condition than in the mother condition, and this type of sympathy and empathy with the victims could contribute to significantly challenging the PI.

### Functional Connectivity

In the stranger condition, several areas exhibit activation with stronger functional connectivity with the seed regions of the AI or PI. For example, the connectivity between the bilateral PFC and AI was stronger in the mother condition, which was consistent with our speculation that the mother condition required higher levels of integration (Wright et al., 2011) and modulation (Decety and Michalska, 2010), including the ones for disgust aversion (Chang et al., 2011) and related negative moral verdicts (Schaich Borg et al., 2011).

However, in the stranger condition, more areas showed stronger functional connections with the PI or AI seed regions. First, stronger connectivity was observed between the thalamus and the PI, which was consistent with the observation that the PI receives input from the thalamus and implies that the moral violation of the stranger evoked a more basic form of disgust. Second, the stronger connectivity between the amygdala and the PI seed region in the stranger condition was also consistent with the higher level of disgust reported by the participants in the stranger condition. In contrast to the connectivity with the thalamus and amygdala, the seed region showing stronger connectivity with the ACC was located in the AI rather than in the PI. This finding was consistent with the hypothesis that the co-activation of the ACC, amygdala, caudate, ventral striatum and the AI represented emotion and motivation values (Craig, 2002). However, the present study only found enhanced connectivity between the amygdala and PI but not with the AI, and both the PI and AI seed regions were increasingly connected to the caudate in the stranger condition. Therefore, our results were only partially consistent with the abovementioned hypothesis (Craig, 2002). Finally, the TPJ, which is among the most important areas for ToM and moral judgment, was more functionally connected with both the PI and AI seed regions in the stranger condition. As previously mentioned, in the stranger condition, the participants could be more likely to perceive themselves as the victims of the moral violations, which may lead to more sympathy and empathy processes that not only evoke activation in the PI but also result in enhanced connectivity between the TPJ and posterior and anterior portions of the insula.

The results of the insula’s functional connectivity with other brain regions were also consistent with the behavioral results in which the RTs in the mother condition were shorter than those in the stranger condition. First, the insular seed region had more functional connectivity with other brain areas in the stranger condition than in the mother condition, suggesting that wider and more extensive information processing (and maybe longer RTs) occurred in the former condition. Second, the stranger condition exhibited more functional connectivity between the insula (including both the AI and PI) and TPJ than did the mother condition, implying that the stranger condition relied more heavily on reasoning processes based on ToM that might have taken longer time. Third, in the mother condition, the AI had stronger connectivity with the PFC, whereas in the stranger condition, the PI had stronger connectivity with the amygdala and thalamus. One possible explanation for this difference is that the enhanced connectivity between the insula and PFC in the mother condition could be related to inhibitory processes caused by the individuals’ reluctance to think negative thoughts about their mother, and this inhibitory process might prevent individuals from further processing the sentences about their mothers.

In this study, although the processing of the materials evoked complicated feelings, emotions and cognitive processes, the feeling of moral disgust could be essentially involved in this complicated processing. Due to its well-established role in disgust, the insula could play a key role in representing moral disgust. However, in the present study, we could not completely justify that the observed insular activation did represent moral disgust rather than other feelings or thoughts. We did not find a significant correlation between the insular activation and individual subjective evaluations of disgust toward the immoral events. A possible interpretation is that the subjective evaluation of moral disgust is a holistic impression consisting of complicated cognitions, emotions, experiences, and social attitudes toward the transgression event. The element of disgust represented by the insula was not sufficiently strong to be reflected by this subjective evaluation. Further studies should adopt specific judgments that are more sensitive to detect the disgust element in moral judgment and verify the role of the insula in disgust representation.

In summary, in this study, we doubly dissociated two insular components in the processing of moral transgression events, and the component located in the posterior region was more activated in the stranger condition, while the other component located in the anterior region was more activated in the mother condition. Given that both the PI and AI were positively activated in the mother and stranger conditions (the signal change in the AI and PI regions was positive in both conditions), we propose that these two components may have been generally involved in both conditions regardless of the behavioral agent of the moral transgression (mother or stranger), and the double dissociation between the AI and PI implies that the stranger and mother conditions could rely on one of the two components more than the other. Based on the already known function of the PI and AI in emotion representations and re-representation and the consideration of the distinctive moral emotions involved in stranger and mother conditions, we hypothesize that the PI and AI might represent primary and secondary levels of moral disgust, respectively. Specifically, the PI component represents people’s basic moral disgust that is directly embodied by the sensory components of physical disgust, whereas the AI components represent a secondary level of moral disgust that is related to the affective components of physical disgust. This result demonstrated the mechanism of embodied schemata from a cognitive neuroscience perspective and showed how disgust in response to moral violations is built on more basic types of disgust (such as the disgust associated with distaste for food and body waste products) and how it develops a more integrative and abstract form of mental representation.

## Ethics Statement

This study was carried out in accordance with the recommendations of Institutional Review Board of the State Key Laboratory of Cognitive Neuroscience and Learning at Beijing Normal University with written informed consent from all subjects. All subjects gave written informed consent. The protocol was approved by the Institutional Review Board of the State Key Laboratory of Cognitive Neuroscience and Learning at Beijing Normal University.

## Author Contributions

All authors listed have made a substantial, direct and intellectual contribution to the work, and approved it for publication.

## Conflict of Interest Statement

The authors declare that the research was conducted in the absence of any commercial or financial relationships that could be construed as a potential conflict of interest.

## Supplementary Material

FIGURE S1 Reaction time, disgust rating, and severity rating in all three conditions: Stranger, Best Friend, and Mother.

TABLE S1 Statistical analysis of moral disgust materials in the three groups.
