# Supplementary figures and images for: Functional Dissociation of the Posterior and Anterior Insula in Moral Disgust
Source: Front Psychol. 2018 Jun 1;9:860. doi: 10.3389/fpsyg.2018.00860 (PMC5992674; doi:10.3389/fpsyg.2018.00860)

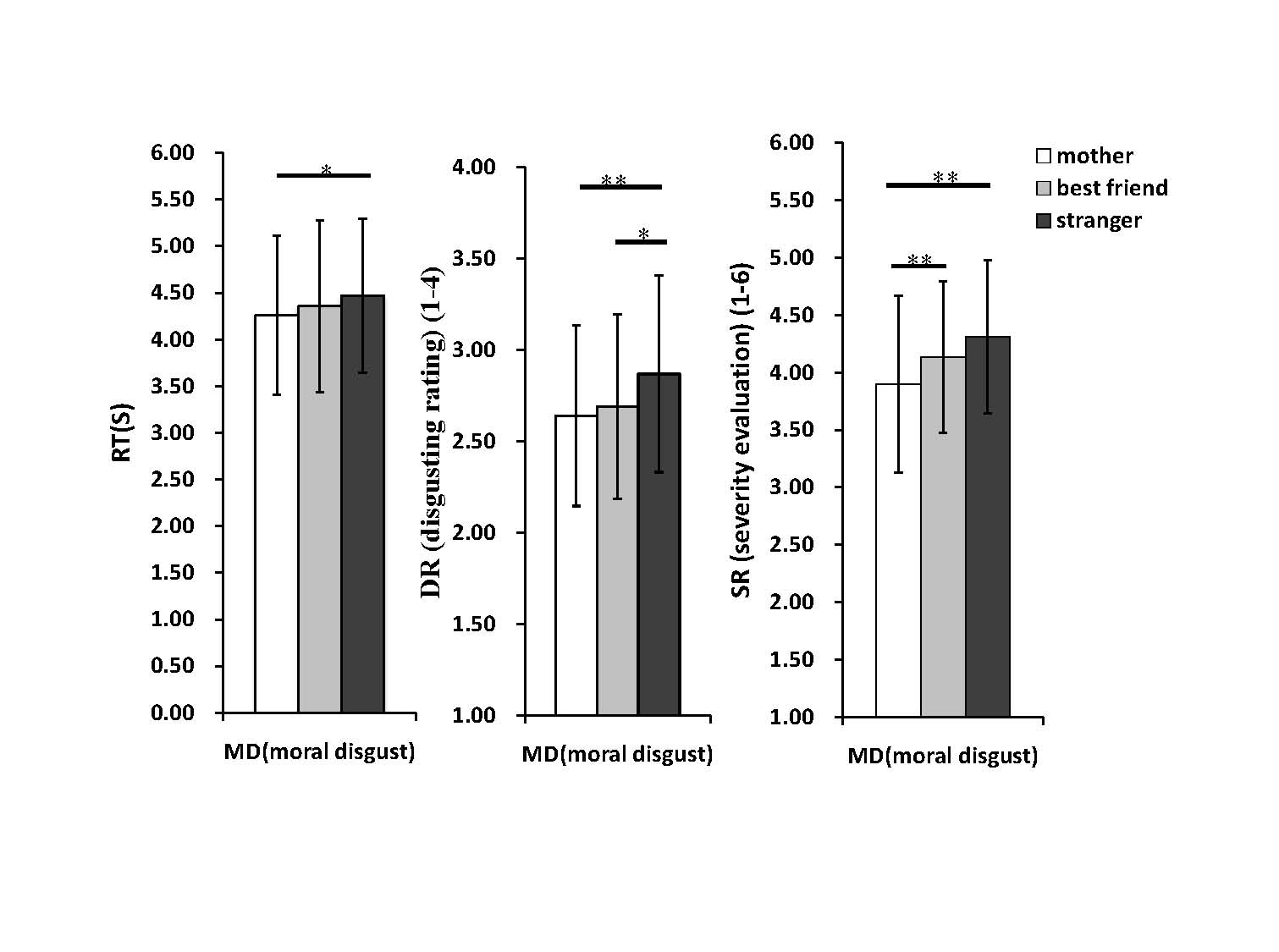

Supplement: FIGURE S1 — Reaction time, disgust rating, and severity rating in all three conditions: Stranger, Best Friend, and Mother. [file Image_1.JPEG]
