# Supplementary material for: Functional Dissociation of the Posterior and Anterior Insula in Moral Disgust
Source: Front Psychol. 2018 Jun 1;9:860. doi: 10.3389/fpsyg.2018.00860 (PMC5992674; doi:10.3389/fpsyg.2018.00860)
Supplement: TABLE S1 — Statistical analysis of moral disgust materials in the three groups. [file Presentation_1.pdf]

Supplementary Material

Tables

Table S1: Statistical analysis of moral disgust materials in the three groups

	**Group 1**<br>Mean<br>(SD)	**Group 2**<br>Mean<br>(SD)	**Group 3**<br>Mean<br>(SD)	F(df)
Length of Sentence	9.85	10.05	10.05	0.115(2,18)
	(1.73)	(1.36)	1.54	ns.
Disgust	4.06	4.05	4.06	0.068(2,98)
	(1.14)	(1.26)	1.26	ns.
Negative Emotion	3.67	3.72	3.72	1.193(2,98)
	(1.01)	(1.16)	1.14	ns.
Severity	4.52	4.50	4.52	0.197(2,98)
	(0.72)	(0.76)	0.73	ns.

Note: Negative Emotion included the following five emotion items: disgust, anger, surprise, sadness, and disappointment.
No significant differences were observed among the 3 groups in length of sentence, disgust rate, negative emotion score, severity of materials by a one-way ANOVA analysis.

Figure Legends

Figure S1. Reaction time and disgust rating and severity rating in all three conditions: Stranger, Best Friend and Mother
